# Research on the Material Compatibility of Elastomer Sealing O-Rings

**DOI:** 10.3390/polym14163323

**Published:** 2022-08-16

**Authors:** Miroslav Müller, Vladimír Šleger, Jakub Čedík, Martin Pexa

**Affiliations:** 1 Department of Material Science and Manufacturing Technology, Faculty of Engineering, Czech University of Life Sciences Prague, Kamycka 129, 165 00 Prague-Suchdol, Czech Republic; 2 Department of Mechanical Engineering, Faculty of Engineering, Czech University of Life Sciences Prague, Kamycka 129, 165 00 Prague-Suchdol, Czech Republic; 3 Department of Quality and Dependability of Machines, Faculty of Engineering, Czech University of Life Sciences Prague, Kamycka 129, 165 00 Prague-Suchdol, Czech Republic

**Keywords:** elastomer seals, O-ring, fuel, polymer application, compatibility, degradation, life cycle assessment

## Abstract

Significant attention has been paid to combustion engines for the utilization of new liquid fuels and their testing at the present. Research activities in ensuring the optimum function of the engine by watching sealing and distribution rubber elements, which are part of fuel systems, should be an integral part of fuels research. When evaluating fuels utilization in combustion engines, the issue has to be judged in a complex. However, when using biofuels in combustion engines, it is not always simple owing to the different degradation properties of these products. Elastomer material is not entirely resistant to various types of fuels. More or less, it is possible to expect changes in its mechanical properties. For the evaluation of the functionality of elastomer sealing elements based on ACM, HNBR and FVMQ type O-rings with pure and blended fuels, the evaluation of changes in mass, hardness Shore A, permanent deformation *CS*, tensile strength *TS* and deformation *Eb* after immersion with the tested fuel is mainly used. Permanent changes were found by the tests. The degradation of elastomer O-rings was more pronounced for the tested fuels containing ethanol, iso-butanol, n-butanol, methanol and dodecanol. HVO 100 fuel containing hydrotreated vegetable oil did not show significant degradation of elastomer O-ring seals. Of the O-rings tested, the FVMQ type O-rings showed the best performance in terms of material compatibility for all fuels tested.

## 1. Introduction

There is a huge number of elastomer materials that can be used in fuel systems. The producers of these elastomer seals define the options and limits of their applications. New elastomer materials for a certain application, namely, composite-based nanoparticle fillers, have been constantly developed [1,2]. Although many decades have passed since the first practical application of the vulcanization of rubber, intensive research in the area of sophisticated methods affecting the chemical process of vulcanization is still underway [3]. Conclusions from computer-aided design and associated mathematical modeling are particularly important for practical applications, especially among manufacturers [3,4].

Significant research has been conducted on the options of new biofuels and their various combinations in the field of fuels. These research activities influence the properties and emissions of combustion engines. It is necessary to not forget the weak points of these engines, and they are elastomer seals and fuel tubes which can have, in the case of the damage or the deterioration of mechanical properties, major consequences for the whole car. So, the development of reliable, high-performance elastomer materials will continue to play a key role in developing new emission-decreasing fuels [1].

Alternative fuels are becoming increasingly popular for use in internal combustion engines. However, many questions still remain regarding the compatibility of these fuels with elastomeric materials [5].

Elastomers are one of the main material groups of sealing elements in fuel systems [6]. They show a good elasticity and chemical resistance. Raw rubber needs a curing process in order to obtain the crosslinking of the molecular chains and provide good final elastomeric mechanical properties. This is an essential process to which it is necessary to pay the same attention as the attention paid to the influence of the individual components of the rubber recipe [7]. The good elasticity of sealing elements can be eliminated by an influence of various fuels [8]. The elastomer material used in fuel systems is not entirely resistant to fuels, and a change in mechanical properties can be expected, namely, by biofuels acting [8].

The influence of common fuels which have been used for many years is relatively well known. At present, new fuels and their various combinations have been created. An O-ring, which has a double-acting sealing effect, is one of basic and the most often used sealing elements [9]. A reproduction of operating conditions is difficult in O-rings testing because of the fact that different results have been often reached due to the different sizes of testing O-rings [9,10]. For example, the results of Bafna [9] can be mentioned, who proved the significance of the dimensional factor of tested samples. The O-ring enables a sealing effect of the system at compression in a radial or an axial direction. A sealing force is created by pressing [8]. A choice of suitable elastomer seals is not simple. It depends on the type of operating medium (a fuel), the temperature and the resultant pressure [11]. A factor of the gradual degradation of the elastomer seals also has to be taken into account. Polyacrylate Rubber (ACM), Chloroprene Rubber (CR), Ethylene Propylene Diene Rubber (EPDR) Fluorocarbon Rubber (FKM), Hydrogenated Acrylonitrile-Butadiene Rubber (HNBR), Acrylonitrile-Butadiene Rubber (Nitrile Rubber) (NBR) and Silicone Rubber (VMQ) can be mentioned as elastomer materials used for seals [11].

Global trends in internal combustion engines have led to the utilization of new biofuels and their various combinations [12,13,14,15,16]. A functionality solution of the fuel system as a whole—namely, in its weakest place, which is elastomer seals and fuel tubes—is an integral part of these research activities. These elastomer parts of the fuel system are exposed to degradation connected with a change in mechanical properties, which leads to their function loss [1,10].

Elastomer products are exposed under the above-mentioned conditions to fatigue cracks and a change in homogeneity influencing the change in mechanical properties [1,17]. Such mechanical damage limits their original functions and can result in serious problems. The danger is caused by a function loss of the elastomer products, on the one hand, but also by the direct impact on safety, namely, when they are used as elastomer-based seals and fuel tubing. Alvina and Borsini [1] state that the suitability of elastomer materials for automotive industry applications has to be tested by a number of various measurements.

It is not possible to use the general recommendations of producers for elastomer materials in fuel systems, namely, in a case where there are various ratios of biofuels as a result. The study by Šleger et al. [6] showed different ratios of rapeseed methyl ester and diesel fuel.

Material degradation is undesirable, and knowledge of the conditions under which the degradation occurs and the means of its protection are very valuable pieces of information from the user’s point of view. For that reason, laboratory testing is important.

Problems with the long-term focus on searching for fuels have become global with climate change and the depletion of fossil fuels [2]. An escape of the fuels from the fuel system is a significant current and future problem [2]. This problem is mitigated by using elastomer seals (O-rings), which, however, degrade owing to these media and the operation environment [2].

Functional sealing elastomer elements are necessary for securing fuel system functionality [10]. Elastomers are one of the main material groups used for the production of seals. An increasing representation of biofuels and different ratios in combustion engines can be expected in the future [18]. However, these fuels and their various combinations can aggressively act as elastomer seals and distribution tubes of fuel systems [1]. So, it is necessary to determine which changes can occur in the long term, which generally increase the degradation of the elastomer material [12,16]. It is necessary to determine the compatibility of elastomer elements exposed to the influence of liquid biofuels in combustion engines with an emphasis on their service life and on keeping the required mechanical properties.

The degradation of rubber materials in biofuels is caused by the presence of various additives in biofuels and rubber blends, oxidation and rubber damage [19].

In an automotive system, a number of modular units, such as the fuel pump, the fuel injectors, the engine and the exhaust system, are in direct contact with the fuel and the sealing rubber element [19]. Since conventional automotive fuel systems have been adapted to petroleum-based fuels, switching to biofuels can potentially cause the swelling and degradation of the rubber [20].

Research shows that the most significant deterioration is in rubber materials compared to various metals and their alloys, which causes swelling and degradation, particularly of O-rings [21].

The rubbers chosen include acrylonitrile butadiene rubber (NBR), fluoroelastomer (FKM), polychloroprene rubber (CR), silicone rubber (SR), hydrogenated NBR (HNBR), ethylene-propylene-diene rubber (EPDM), acrylic rubber (ACM), epichlorohydrin (ECO), styrene butadiene rubber (SBR) and polyurethane, which are very often used to make O-rings in fuel systems [19].

The compatibility of individual elastomer materials (abbreviations according to ISO 1629) and the resistance to liquids that can be used in the automotive industry are presented in Table 1 [11]. However, the authors of this publication state that the presented resistance and compatibility values of individual elastomer materials are orientations, and they always have to be experimentally verified because of the various physical aspects of a specific application in the fuel system [11]. However, it is possible to regard this table as a basic presumption narrowing the selection of tested materials.

Table 1 summarizes the changes in the properties of the investigated rubbers after exposure to different biofuels.

The aim of this research was the long-term monitoring of the compatibility of elastomer O-ring-based seals with pure and blended fuels and the associated definition of mechanical tests, i.e., the material compatibility of elastomer seals and fuel was evaluated on the basis of permanent deformation *CS*, tensile strength *TS*, elongation *Eb*, hardness Shore A and mass.

## 2. Materials and Methods

The material compatibility of the elastomer seal (sealing O-rings) and the fuel is tested over a time interval of 2998 h (approx. 4.2 months, 125 days), which is determined in accordance with ASTM D471-16 (Standard Test Method for Rubber Property Effect of Liquids).

Elastomer O-rings made of Polyacrylate Elastomer—ACM (trade name Nipol AR^®^), Hydrogenated Acrylonitrile Butadiene Elastomer—HNBR (trade name Therban^®^) and Fluorosilicone Elastomer—FVMQ (trade name Silastic^®^) were used for the research. The supplier of the elastomer O-rings was Bohemia Seal, s.r.o.

According to the supplier’s specification, the O-rings made from ACM are highly resistant against oils with a high temperature; they are used in the automotive industry for sealing the engine and for gearbox oil and other high-temperature lubrication systems. The ACM O-rings have a temperature range from −20 to 175 °C. The O-rings made from HNBR are highly resistant against mineral oils with additives, and their temperature range is from −20 to 150 °C. The O-rings made from FVMQ are highly resistant to oils, fuels and solvents, especially aromatic and chlorinated hydrocarbons and alcohols; their temperature range is from −60 to 200 °C.

The test fuels that were used in the tests aimed at influencing the chemical resistance and compatibility of the elastomer materials defined in Table 2.

Since ethanol and methanol are insoluble in diesel, it is necessary to use co-solvents in order to prevent a phase separation. Iso-butanol and dodecanol were used as the co-solvents. For 5% blends, only iso-butanol was used; for 10% blends, the use of the combination of iso-butanol and dodecanol was necessary, especially for methanol. For fuel blends with n-butanol, no co-solvent was used, as there are no solubility problems with n-butanol in diesel, and no phase separation was observed.

Specifications of the test fuels and their base components are shown in Table 3. The fuel density and kinematic viscosity were determined by means of the Stabinger Viscometer SVM 3000 from Anton Paar GmbH (measurement accuracy < 1%, repeatability = 0.1%).

The calorific values of the base fuels were determined using the isoperibol calorimeter LECO AC600 (range 23.1–57.5 MJ/kg for a 0.35 g sample, accuracy 0.1% RSD) according to CSN DIN 51900-1 [22] and CSN DIN 51900-2 [23].

**Table 3:** Basic specifications of the tested fuels.

Fuel	Kinematic Viscosity at 40 °C (mm^2^ s^−1^)	Density at 15 °C (kg m^−3^)	Calorific Value (MJ kg^−1^)	Cetane Number	Carbon Content (% wt)	Hydrogen Content (% wt)	Oxygen Content (% wt)
D100	1.878	820.67	43.2	50 [24]	87 [24]	13	0
M100	0.563	797.57	19.6	<5 [25]	37.5	12.6	49.9
E100	1.21	812.93	26.8	5–8 [26]	52.2	13.1	34.7
B100	2.266	815.27	33.1	17–25 [27,28]	64.8	13.6	21.6
HVO100	2.905	781.87	44	>75 [29]	85 [30]	15	0
B10	1.765	819.2	42.2	-	84.8	13.1	2.1
B20	1.751	818.4	41.2	-	82.7	13.1	4.2
B30	1.773	817.7	40.2	-	80.4	13.2	6.4
E5 ISO BUT	1.737	818.2	41.9	-	84.2	13	2.8
M5 ISO BUT	1.688	817.8	41.6	-	83.6	13	3.4
E10 ISO BUT	1.71	816.7	40.6	-	81.4	13.1	5.5
M10 2.5DOD, 6Isobut	1.655	816.5	40.3	-	80.7	13	6.3
E10 2.5DOD, 6Isobut	1.775	817.9	40.9	-	82	13.1	4.9
Iso-butanol	2.729	807.17	33.1	<15 [31]	64.8	13.6	21.6
Dodecanol	11.412	828.87	39.9	63.6 [32]	77.3	14.1	8.6

The mass contents of the carbon, hydrogen and oxygen of the alcohols were calculated based on the atom masses of the atoms in their molecule. For diesel, the content is given by EN 590, and for HVO, it is given by EN 15940. The C, H and O contents of the fuel blends were calculated based on the mass concentration of the individual alcohols in the blends, which was determined using the measured densities of the base fuels and their volumetric concentrations.

The material compatibility of the elastomer seal and the fuel was evaluated based on permanent deformation *CS* (%), tensile strength *TS* (MPa), elongation *Eb* (%), hardness Shore A SHA and mass before and after immersion in the tested fuel. Material compatibility measurements after the removal of the O-rings from the test fuels were performed after the evaporation of the volatile fuel in accordance with ASTM D471-16, which was determined by stopping the mass loss of the samples on the analytical scales.

The measurements of permanent deformation *CS* (%), tensile strength *TS* (MPa) and elongation *Eb* (%) were carried out on the electromechanical testing machine MPTest 5.050 of the Czech company LaborTech. According to EN 7500-1, the device meets the requirements for class 0.1. The accuracy of the force gauge used is 0.1 N; the position of the crossbar is detected with an accuracy of 0.001 mm.

The permanent deformation–compression set is another essential parameter for determining the sealing properties of the elastomers in the fuel systems. The fact that pressing causes not only their elastic deformation but also their plastic deformation is a reason for this. The permanent deformation *CS* can considerably differ owing to the degradation acting of the liquid contaminant fuels.

**Figure 1:**
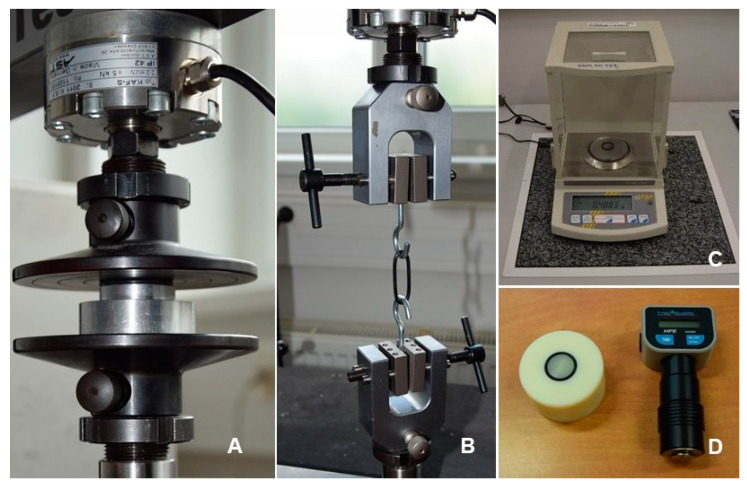
Measurement of the mechanical properties: (**A**): permanent deformation *CS*, (**B**): tensile strength *TS* and elongation *Eb*, (**C**): mass, (**D**): hardness Shore A SHA.

The permanent deformation *CS* was measured according to the modified standard CSN ISO 815-1 (Rubber, vulcanized or thermoplastic elastomer—Determination of permanent deformation in compression—Part 1: At laboratory or elevated temperatures). The research results of Muller et al. [6] proved the possibility of testing O-rings from the fuel system. The modification of the standard consisted in the use of real sealing rings and the loading time. The measurement of the permanent deformation of the O-rings can be seen in Figure 1A.

The permanent deformation *CS* can be calculated according to Equation (1), where: *CS*—permanent deformation-compression set (%), *h*_0_—initial height of the O-ring (mm), *h*_1_—height in the state of compression (mm), *h*_2_—height after release (mm).

(1) $$CS=\frac{h_{0}-h_{2}}{h_{0}-h_{1}}\times 100\%$$

The tensile strength *TS* and the elongation Eb can be determined in accordance with the standard. The tensile properties of the rubber were determined according to the modified standard ČSN ISO 37 (Rubber, vulcanized or thermoplastic elastomer—Determination of tensile properties). The modification consisted in testing real fuel seals. The modification of real products determined for the fuel systems was proved by the scientific studies of Muller et al. [6]. For example, the authors recommend using hooks for O-rings clamping to prevent damage of the surface integrity and decreases in the tested material strength.

The tensile strength *TS* can be calculated according to Equation (2), where: *TS*—tensile strength (MPa), *F_m_*—maximum tensile force (N), *h*_0_—initial height of the O-ring (mm).

(2) $$TS=\frac{F_{m}}{\left(\frac{2\pi h_{0}^{2}}{4}\right)}$$

The elongation *E_b_* can be calculated according to Equation (3), where: *E_b_*—elongation (%), *C_b_*—final inner circumference of the O-ring (mm), *C_j_*—initial inner circumference of the O-ring (mm).

(3) $$E_{b}=\frac{(C_{b}-C_{j)}}{C_{j}}\times 100\%$$

The initial inner circumference of the O-ring *C_j_* was calculated from the inner diameter of the O-ring, which was determined from the distance of the hooks during the tensile test, when the measured force started to increase. The final inner circumference of the O-ring *C_b_* was calculated from Formula (4), where 2.9 mm is the hook wire diameter.

(4) $$C_{b}=(\pi \times 2.9)+(2\times 2.9)+(2\times L_{b})$$

where: *C_b_*—final inner circumference of the O-ring (mm), *L_b_*—distance of the hooks when the O-ring breaks (mm).

The tensile strength and elongation measurements of the O-rings can be seen in Figure 1B.

The initial mass of the specimen (sealing O-rings) in air and the mass of the specimen in air after the immersion in the tested fuel were determined by weighing on KERN analytical scales. The measurement of the mass of the O-rings can be seen in Figure 1C.

The hardness is the most often tested mechanical quantity of elastomer materials. The hardness can be defined as the body resistance to the penetration of another harder body (indenter) into a tested surface. However, the measurement of the elastomer material hardness is a problem owing to the thickness of the tested material. The standard CSN EN ISO 868 states a minimum value of 4 mm for the Shore A method. It is suitable to use this method (Shore A) for elastomer seals because it is determined for soft to medium-hard elastomer materials. It is possible to solve this problem by layering several elastomer materials on top of each other, which the standard enables. The authors Muller et al. introduced a special device for testing layered O-rings hardness [6]. The measurement of the hardness Shore A of the O-rings can be seen in Figure 1D.

Figure 1 shows the testing of the initial mechanical properties of the O-rings and the properties after a specified time of 2998 h after their immersion in the tested fuels. The material compatibility of the elastomer seal and fuel based on permanent deformation *CS* (%) can be seen in Figure 1A; the tensile strength *TS* (MPa) can be seen in Figure 1B; the elongation *Eb* (%) can be seen in Figure 1B; the mass can be seen in Figure 1C; the hardness Shore A SHA can be seen in Figure 1D.

The change in the mechanical properties of permanent deformation *CS* (%), tensile strength *TS* (MPa), elongation *Eb* (%), hardness Shore A SHA and mass before and after immersion in the tested fuels is calculated as a percentage change and is in accordance with ASTM D471-16.

The percentage change in mass Δ*M* was calculated using Formula (5), where: Δ*M*—change in mass (%), *M*_0_—initial mass of the O-ring specimen in air before immersion in the fuel (mg), *M*_1_—mass of the O-ring specimen in air after immersion in the fuel (mg).

(5) $$\Delta M=\frac{\left(M_{1}-M_{0}\right)}{M_{0}}\times 100$$

The change in hardness Δ*H* after immersion is given in units of hardness and is calculated according to Formula (6), where: Δ*H*—hardness change after immersion (ShA), *H*_0_—initial hardness of the O-ring specimen in air before immersion in the fuel (ShA), *H*_1_—hardness of the O-ring specimen in air after immersion in the fuel (ShA).

(6) $$\Delta H=H_{1}-H_{0}$$

The percentage change in tensile strength Δ*TS* was calculated according to Formula (7), where: Δ*TS*—change in tensile strength (%), *TS*_0_—initial tensile strength of the O-ring specimen in air before immersion in the fuel (%), *TS*_1_—tensile strength of the O-ring specimen in air after immersion in the fuel (%).

(7) $$\Delta TS=\frac{\left(TS_{1}-TS_{0}\right)}{TS_{0}}\times 100$$

The percentage change in elongation Δ*Eb* was calculated according to Formula (8), where: Δ*Eb*—change in elongation (%), *Eb*_0_—initial elongation of the O-ring specimen in air before immersion in the fuel (%), *Eb*_1_—elongation of the O-ring specimen in air after immersion in the fuel (%).

(8) $$\Delta Eb=\frac{\left(Eb_{1}-Eb_{0}\right)}{Eb_{0}}\times 100$$

The percentage change in permanent deformation Δ*CS* was calculated according to Formula (9), where: Δ*CS*—change of permanent deformation (%), *CS*_0_—initial permanent deformation of the O-ring specimen in air before immersion in the fuel (%), *CS*_1_—permanent deformation of the O-ring specimen in air after immersion in the fuel (%).

(9) $$\Delta CS=\frac{\left(CS_{1}-CS_{0}\right)}{CS_{0}}\times 100$$

## 3. Results and Discussion

The compatibility of the tested fuels and their blends with the ACM, HNBR and FVMQ O-rings was investigated based on changes in the mechanical properties of permanent deformation Δ*CS*, tensile strength Δ*TS*, elongation Δ*Eb*, hardness Shore A Δ*H* and mass Δ*M*.

Higher initial values of permanent deformation *CS* are exhibited by the ACM (0.13 ± 0.01) and HNBR (0.14 ± 0.01) O-rings compared to FVMQ (0.10 ± 0.01), while tensile strength TS is exhibited by the HNBR O-rings (30.1 ± 4.03 MPa) compared to ACM (12.18 ± 0.94 MPa) and FVMQ (15.21 ± 0.55 MPa). The FVMQ O-rings (3.27 ± 0.10) exhibit significantly higher elongation values *Eb* compared to ACM (1.32 ± 0.08) and HNBR (1.60 ± 0.18). The HNBR O-rings (71.30 ± 2.05 ShA) exhibited significantly higher hardness Shore A values compared to ACM (50.14 ± 2.09 ShA) and FVMQ (42.82 ± 0.23 ShA).

**Figure 2:**
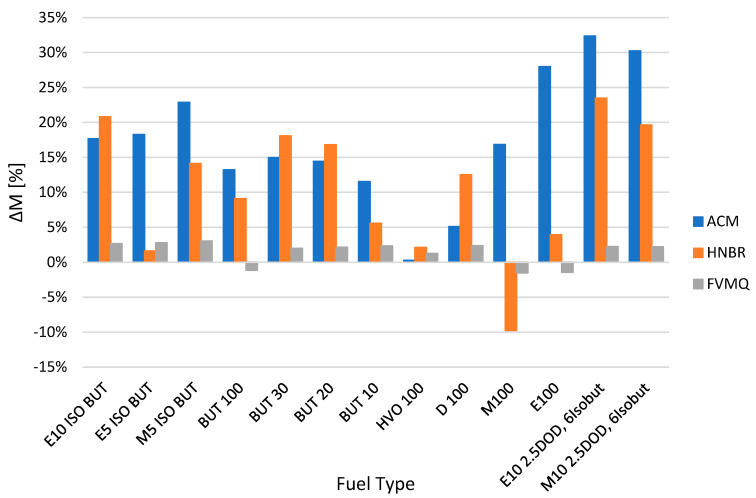
Change in mass Δ*M* for different elastomer O-rings and fuels after exposure for a time interval of 2998 h.

Figure 2 shows the change in mass Δ*M* for different elastomer O-rings and fuels after exposure for a time interval of 2998 h. Figure 2 shows the increase and decrease in the mass of elastomer O-rings after exposure in different types of fuels.

The results show that the FVMQ sealing O-ring has good compatibility with the tested fuels in terms of mass loss. There was change in mass Δ*M* ranging from −1.5% to 3%. The change in mass was almost constant compared to the other materials tested, which showed a change in mass Δ*M* of −9.7% to 32.4%.

On the other hand, the O-rings made of ACM and HNBR are less compatible. The results show a high mass loss for the fuel M100 at the HNBR O-rings. Conversely, the results show a high mass gain at the ACM O-rings in the fuels M5 ISO BUT, E100, E10 2.5DOD 6Isobut and M10 2.5DOD 6Isobut.

The swelling of the O-rings can be represented by changing their mass and the initial mass of the O-ring specimen in air before immersion in the fuel to the mass of the O-ring specimen in air after immersion in the fuel [33]. An increase in mass after the degradation process was observed for the ACM and HNBR O-rings, except for the fuel M100, where there was a decrease in the mass of the O-ring specimen in air after immersion in the fuel from HNBR. From the results, it can be concluded that the fuel HVO had a minimal effect on the change of all three O-ring materials tested. In general, the increase in mass is related to the swelling of elastomers, which is caused by an absorption of the fuel into the polymer chains [34].

**Figure 3:**
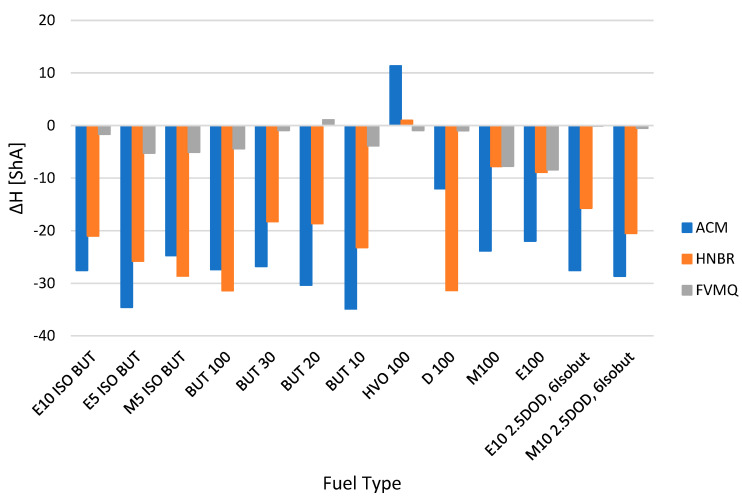
Change in hardness Shore A Δ*H* for different elastomer O-rings and fuels after exposure for a time interval of 2998 h.

**Figure 4:**
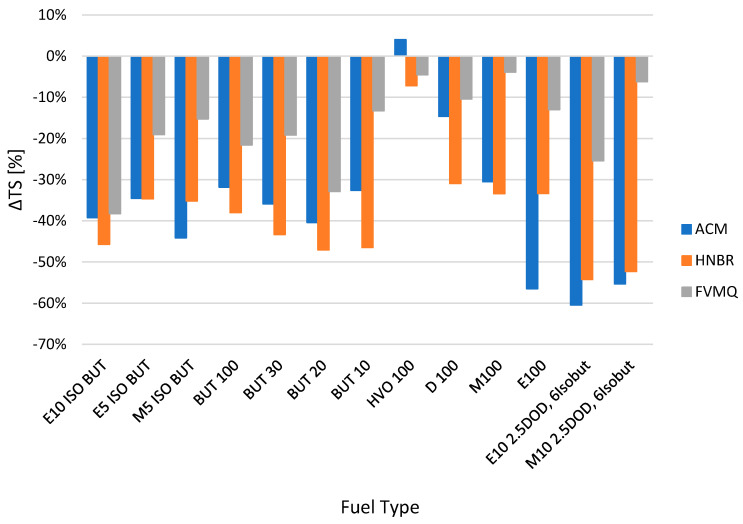
Change in tensile strength Δ*TS* for different elastomer O-rings and fuels after exposure for a time interval of 2998 h.

**Figure 5:**
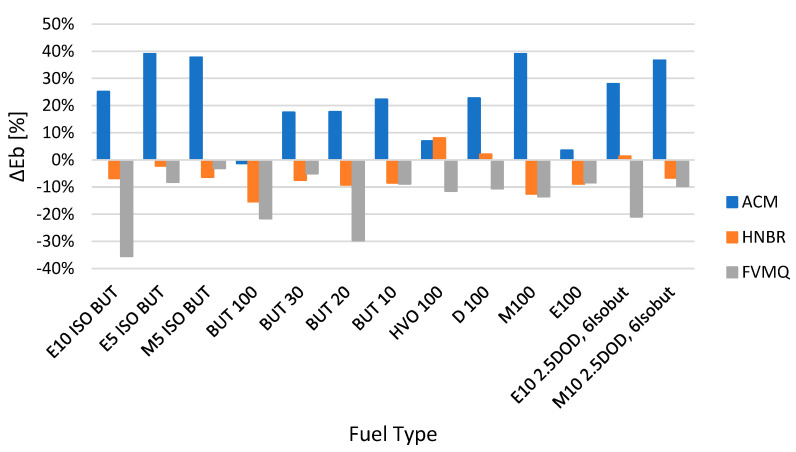
Change in elongation Δ*Eb* for different elastomer O-rings and fuels after exposure for a time interval of 2998 h.

**Figure 6:**
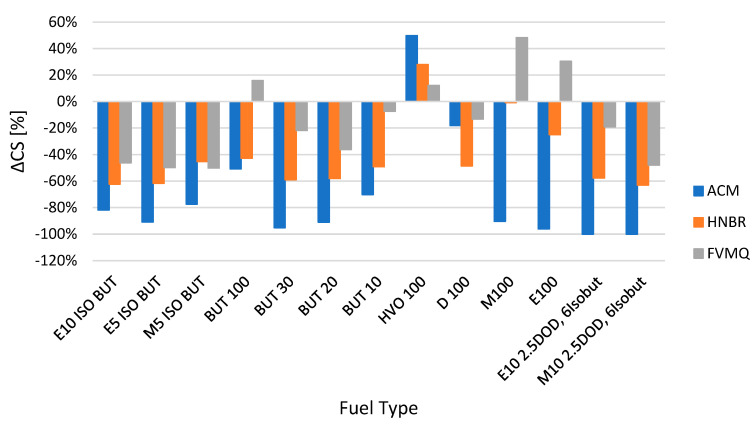
Change in permanent deformation Δ*CS* for different elastomer O-rings and fuels after exposure for a time interval of 2998 h.

The influence of the change in mechanical properties is evident from the other figures, i.e., hardness Shore A Δ*H* (Figure 3), tensile strength Δ*TS* (Figure 4), elongation Δ*Eb* (Figure 5) and, last but not least, permanent deformation Δ*CS* (Figure 6).

Figure 3 presents the change in hardness Shore A Δ*H* for different elastomer O-rings and fuels after exposure for a time interval of 2998 h. Figure 3 presents the hardness Shore A loss of elastomer O-rings after exposure in different types of fuels. The HVO fuel, which did not show a decrease in hardness Shore A, exhibited a different behavior among the tested fuels. On the other hand, there was a slight increase of 11.32% for the ACM sealing O-rings and of 0.96% for the HMBR sealing O-rings. For the elastomer O-rings of type FVMQ, which were tested last, there was a slight decrease in hardness not exceeding 1%.

The results show that the FVMQ O-ring has good compatibility with the tested fuels in terms of eliminating significant variations in hardness at the interval of −8.42 to 1.08%. Figure 3 shows significant changes in hardness Shore A approaching 35%. Hardness reduction is also presented by other researchers in their publications on the compatibility of different elastomers with diesel and different biodiesel blends [33,35]. Carbon black and silica fillers, which primarily serve to improve hardness and tensile properties, can be considered as the cause of the hardness decrease in various biofuels. These components can react with biofuels after a certain exposure time and, secondarily, can deteriorate the above-mentioned properties [33].

Figure 4 shows the change in tensile strength Δ*TS* for different elastomer O-rings and fuels after exposure for a time interval of 2998 h. From the results, it is clear that almost all the tested fuels and elastomer O-rings lose tensile strength TS in the interval from −60.4 to −3.9%. Smaller changes in tensile strength *TS* can be observed for fuel HVO100 at all three sealing elastomer O-rings. However, the FVMQ sealing O-rings showed smaller changes compared to the ACM and HNBR sealing O-rings.

There was a slight increase in tensile strength *TS* of 4.0% for elastomer O-rings of type ACM placed in HVO 100 fuel.

Significant changes in tensile strength *TS* exceeding 60% were exhibited by some fuels, as can be seen in Figure 4—particularly, fuels designated as E100, E10 2.5DOD, 6Isobut and M10 2.5DOD 6Isobut and elastomer O-rings of the ACM and HNBR types.

This was an approximately similar trend of changes in ∆*TS* as the trend in the loss of hardness ∆*H* (ShA), which has been referred to in other papers focusing on similar research [36].

Figure 5 shows the change in elongation Δ*Eb* for the different elastomer O-rings and fuels after exposure for a time interval of 2998 h. The results show a decrease in elongation Δ*Eb* for all of the tested fuels for the elastomer FVQM O-ring and for most of the tested fuels, except for HVO100, D100 and E10 2. 5DOD 6Isobut, for elastomer O-rings of type HNBR. A significant increase in elongation Δ*Eb* was found for almost all of the tested fuels, except for the fuel BUT 100 (decrease of −1.3%), for elastomer O-rings of type ACM. In particular, these O-rings showed a significant change in shape and elongation Δ*Eb* of up to about 40%, which did not allow for their possible use. These results correspond to a significant change in Δ*M*.

Figure 6 shows the change in permanent deformation Δ*CS* for different elastomer sealing O-rings and fuels after exposure for a time interval of 2998 h. From the experimental results shown in Figure 6, it is clear that different fuels mostly reduce the permanent deformation of O-rings. The sealing elastomer O-rings were more elastic. Considering the function of the sealing O-rings, this is a positive effect. The literature on rubber sealing elements indicates that larger permanent deformation values are significantly negative [37]. An increase in the permanent deformation Δ*CS* values occurred for the fuel HVO 100 at all three elastomer sealing O-rings. Furthermore, an increase in the permanent deformation Δ*CS* value occurred at the elastomer O-rings of type FVMQ for the fuels BUT100, M100 and E100.

## 4. Conclusions

From the obtained results, it is possible to draw the following conclusions related to the monitoring of the long-term compatibility of elastomer seals based on O-rings of type ACM, HNBR and FVMQ with pure and blended fuels.

- The mass change due to the effect of the tested pure and blended fuels was minimal for the FVMQ type O-rings. The values were almost constant, with the change in mass Δ*M* ranging from −1.5% to 3%. The change in mass was more pronounced for the ACM and HNBR O-rings, with a change in mass Δ*M* of −9.7% to 32.4%. These O-rings are less compatible with the fuels tested. In particular, the HNBR type O-rings, and especially the ACM type O-rings, experienced significant losses, especially for the fuels M100, M5 ISO BUT, E100, E10 2.5DOD 6Isobut, M10 2.5DOD 6Isobut and E10 ISO BUT.
- Permanent changes were found when evaluating the elastomer O-rings. Mechanical tests of hardness Shore A, tensile strength *TS*, elongation *Eb* and permanent deformation *CS* focused on the changes in their material compatibility in different fuels. The changes in hardness Shore A were mostly manifested by a decrease in their hardness due to time and the exposure to the tested fuels. Only the fuel HVO 100 showed a significant increase in hardness Shore A values. In terms of the tested elastomer rings, the FVMQ type O-ring was shown by measurements to have good compatibility with the fuels tested in terms of eliminating significant variations in hardness changes in the interval from −8.42 to 1.08%. A similar trend of behavior was observed for the tensile strength TS results. From the results, it is clear that almost all of the tested fuels, except for HVO 100 and the elastomer rings ACM, HNBR and FVMQ, lose tensile strength *TS* in the interval from −60.4 to −3.9%. Again, the tensile strength *TS* results showed that the FVMQ O-rings showed less of a change compared to the ACM and HNBR O-rings. The results also showed a significant effect on the change in elongation Δ*Eb*. In particular, elastomer O-rings of the ACM type showed a significant change in shape, and their elongation Δ*Eb* changed up to about 40%. This significant change would not allow for their possible use in a fuel system. The fuels tested also had a significant effect on the changes in permanent deformation Δ*CS* for the different elastomer O-rings, with a reduction in permanent deformation in most tests.
- The degradation of elastomer O-ring seals was also more pronounced for fuels containing ethanol, iso-butanol, n-butanol, methanol and dodecanol. The fuel HVO 100 contains hydrotreated vegetable oil, which did not significantly affect the degradation of elastomer O-ring seals. Of the O-rings tested, the FVMQ type O-rings showed the best performance in terms of material compatibility and the dependence on the fuels tested.

## Figures and Tables

**Table 1:** Chemical resistance and compatibility of elastomer materials with different chemicals (evaluating system—A: very good applicability, no or minimum influence on the change in physical properties; B: very good resistance, a mild change in physical properties; C: limited applicability, it can lead to a significant change in physical properties; D: not recommended for use, unsuitable combination; X: not possible to evaluate) [11].

Chemical Name	ACM	HNBR	FVMQ
Alcohol (methanol)	D	A	A
Aromatic fuels (up to 50% aromatic hydrocarbons)	B	A	D
Petrol	C	A	D
Butanol	D	A	B
Isobutyl alcohol	D	B	A
Methanol	D	B	A
Mineral oil	A	A/B	B
Diesel fuel	D	A	D
Petrol	C	A	D
Engine oil	B	A	D
Oil	B	D	D
Palm oil	A	A	D
Olive oil	A	A	B
Vegetable oils	B	A	B
Rapeseed oil	B	B	D
Air	A	A	A

**Table 2:** Pure and blended tested fuels.

Fuel	Volumetric Composition
E10 ISO BUT	80% diesel, 10% ethanol, 10% iso-butanol
E5 ISO BUT	90% diesel, 5% ethanol, 5% iso-butanol
M5 ISO BUT	90% diesel, 5% methanol, 5% iso-butanol
BUT 100	100% n-butanol
BUT 30	70% diesel, 30% n-butanol
BUT 20	80% diesel, 20% n-butanol
BUT 10	90% diesel, 10% n-butanol
HVO 100	100% hydrotreated vegetable oil
D100	100% diesel
M100	100% methanol
E100	100% ethanol
E10 2.5DOD, 6Isobut	81.5% diesel, 10% ethanol, 6% iso-butanol, 2.5% dodecanol
M10 2.5DOD, 6Isobut	81.5% diesel, 10% methanol, 6% iso-butanol, 2.5% dodecanol

## Data Availability

Not applicable.
